# Risk of seizure recurrence in people with single seizures and early epilepsy – Model development and external validation

**DOI:** 10.1016/j.seizure.2021.11.007

**Published:** 2022-01

**Authors:** Laura J. Bonnett, Lois Kim, Anthony Johnson, Josemir W. Sander, Nicholas Lawn, Ettore Beghi, Maurizio Leone, Anthony G. Marson

**Affiliations:** a Department of Health Data Science, University of Liverpool, Block B, Waterhouse Building, Brownlow Hill, Liverpool L69 3GL United Kingdom; b Cardiovascular Epidemiology Unit, Strangeways Research Laboratory, University of Cambridge, Wort's Causeway, Cambridge CB1 8RN, United Kingdom; c Medical Research Council Clinical Trials Unit, UCL Institute of Clinical Trials and Methodology, London, WC1V 6LJ, United Kingdom; d NIHR University College London Hospitals Biomedical Research Centre, London W1T 7DN, United Kingdom; e UCL Queen Square Institute of Neurology, London WC1N 3BG, United Kingdom; f Chalfont Centre for Epilepsy, Chalfont St Peter, SL9 0RJ, United Kingdom; g Stichting Epilepsie Instelligen Nederland (SEIN), Heemstede 2103 SW, the Netherlands; h Western Australian Adult Epilepsy Service, Perth, Australia; i Department of Neuroscience, Istituto di Ricerche Farmacologiche Mario Negri IRCCS, Milano, Italy; j Fondazione IRCCS Casa Sollievo della Sofferenza, Unit of Neurology, San Giovanni Rotondo (FG), Italy; k Department of Pharmacology and Therapeutics, University of Liverpool, Liverpool, United Kingdom

**Keywords:** ASM, Antiseizure medication, MESS, Multicentre Study of Early Epilepsy and Single Seizures, NGPSE, National general practice study of epilepsy and epileptic seizures, WA, Western Australian study, Independent data, Newly diagnosed, Prognosis, Risk assessment

## Abstract

**Purpose:**

Following a single seizure, or recent epilepsy diagnosis, it is difficult to balance risk of medication side effects with the potential to prevent seizure recurrence. A prediction model was developed and validated enabling risk stratification which in turn informs treatment decisions and individualises counselling.

**Methods:**

Data from a randomised controlled trial was used to develop a prediction model for risk of seizure recurrence following a first seizure or diagnosis of epilepsy. Time-to-event data was modelled via Cox's proportional hazards regression. Model validity was assessed via discrimination and calibration using the original dataset and also using three external datasets – National General Practice Survey of Epilepsy (NGPSE), Western Australian first seizure database (WA) and FIRST (Italian dataset of people with first tonic-clonic seizures).

**Results:**

People with neurological deficit, focal seizures, abnormal EEG, not indicated for CT/MRI scan, or not immediately treated have a significantly higher risk of seizure recurrence. Discrimination was fair and consistent across the datasets (c-statistics: 0.555 (NGPSE); 0.558 (WA); 0.597 (FIRST)). Calibration plots showed good agreement between observed and predicted probabilities in NGPSE at one and three years. Plots for WA and FIRST showed poorer agreement with the model underpredicting risk in WA, and over-predicting in FIRST. This was resolved following model recalibration.

**Conclusion:**

The model performs well in independent data especially when recalibrated. It should now be used in clinical practice as it can improve the lives of people with single seizures and early epilepsy by enabling targeted treatment choices and more informed patient counselling.

## Highlights

• Model predicts risk of seizure recurrence after single fit or epilepsy diagnosis.
• Model performs well in independent data.
• Future work required to ensure the model is adopted in clinical practice.
• Model can improve the lives of people with single seizures and early epilepsy.

## 1 Introduction

A first unprovoked seizure is a common presentation with an estimated incidence of between 50 and 70 per 100,000 in high-income countries [1]. Approximately half will have a seizure recurrence [2], be diagnosed with epilepsy, and will usually start treatment with an antiseizure medication (ASM) to prevent further seizures. ASMs are, however, associated with adverse effects, including teratogenicity. Whilst for most people diagnosed with epilepsy, the benefits of treatment will exceed the risks. This benefit-risk ratio is more finely balanced for those who have had a single seizure. Similarly, the benefit-risk ratio is also more tuned for those who have had two or more seizures with minor symptomatology (*e.g.* focal seizures with retained awareness), or have had long intervals between seizures.

The Multicentre Study of Early Epilepsy and Single Seizures (MESS) considered the benefits of starting or delaying treatment after a first unprovoked seizure and in people with early epilepsy for whom there was uncertainty risk-benefit trade-off of starting ASM [3]. MESS showed that immediate treatment with the commonly used ASM carbamazepine and valproate reduced the risk of further seizures (hazard ratio for time to first seizure 0.7 (95% confidence interval (CI) 0.6– 0.8)) compared to starting treatment after the second or subsequent seizures. There was, however, no evidence of an effect on long-term remission [3]. Analysis of quality of life outcomes showed that the benefit associated with a reduction in seizure recurrence was offset by adverse events and stigma associated with taking an ASM [4].

Data from MESS was used to develop a prediction model estimating the risk of seizure recurrence after a single seizure for people of driving age (aged 16 years or over) [5]. In this model, follow-up started at the first seizure as the intention was to investigate the time interval from that seizure to the time point at which recurrence risk dropped below a threshold that would allow a return to driving. This model was validated in independent data [6] and consequently used to inform driving regulations in the UK and Europe. Evidence was also provided to support one worldwide overall prediction model for risk of second seizure following a first in people of driving age [6].

The MESS data was also used to develop a prediction model for the chance of seizure recurrence for people of all ages, with single seizures and early epilepsy [7]. For this model, follow-up started at the time of the clinic visit at which the diagnosis of seizure was made, on average 12 weeks after the most recent event [7]. A third of participants were recruited within one week of their most recent seizure [3]. This is usually when decisions are made regarding treatment. This is the most informative point from which to measure the subsequent outcome. This model identified significant factors for future seizures, and was used to develop a prognostic index to identify risk groups with associated risk estimates at various time points. This model was developed and validated by dividing the MESS dataset into a test and validation sample in a 6:4 ratio, but was not validated in independent data. Given the availability of relevant independent data, it is now a priority to perform such validation. This divided sample modelling is no longer recommended practice [8]. Consequently, the prediction model for risk of seizure recurrence, for individuals of all ages, with a single seizure or early epilepsy, has been updated according to current recommended statistical methodology [9]. This model is externally validated using three independent datasets – one from the United Kingdom, one from Australia, and one from Italy. This work is vital to ensure that people with single seizures and early epilepsy can make informed decisions regarding treatment choice and receive appropriate counselling.

## 2 Material and methods

### 2.1 Description of studies

#### 2.1.1 Study used for model development

MESS [3] was a UK-based randomised controlled trial that compared immediate or deferred treatment policies in people presenting with a first unprovoked seizure or early onset epilepsy. The clinician and the individual were uncertain about the need for ASM. Recruitment was from 1st January 1993 to 31st December 2000 with final follow-up from 31st December 2001 to 30th June 2002.

Individuals were eligible for inclusion if they were at least one month old; reported at least one clinically definite, spontaneous, unprovoked seizure; and if the clinician and the individual (or carer) were uncertain about whether or not to start ASM. Exclusion criteria included previous treatment with ASMs or the presence of a progressive neurological disease.

Participants were randomly allocated to treatment policy, by phone or fax, using the minimisation method, balanced across centre or region and number of seizures at randomisation. For participants assigned to immediate treatment, the clinician selected the optimum ASM, based on their usual practice and started treatment as early as possible. Participants assigned to deferred treatment received no drugs until the clinician and individual agreed that it was necessary, mainly after a seizure recurrence.

MESS remains the largest reported study of people with single seizures and early epilepsy. While the study's primary purpose was to compare treatment policies, it also provided an important opportunity to examine seizure recurrence risks and factors that modify those risks.

#### 2.1.2 Studies used for validation

*National general practice study of epilepsy and epileptic seizures (NGPSE):* The NGPSE [10] recruited participants between June 1984 and October 1987. It used the UK primary care system to obtain comprehensive data on a large and unselected cohort of people with newly diagnosed seizures, including children with febrile seizures. Individuals were identified for the study via their general practitioner (GP), but were never contacted directly by the study team. The accepted practice for observational studies at the time was that individuals did not need to provide consent as their care was not affected by inclusion in such studies. Over a thousand people were initially referred by 275 GP surgeries, of whom 220 out of 1195 were children with febrile seizures [10]. In NGPSE, the follow-up start date was the date of index seizure, which prompted the GP to register the person in the study.

*Western Australian study (WA):* The WA [11,12] observational dataset included adults referred to the First Seizure Clinics of Royal Perth and Fremantle Hospitals, two major teaching hospitals in Western Australia. Individuals with a first-ever seizure were recruited between January 1999 and December 2015.

People with status epilepticus, prior seizures of any nature except febrile convulsions, and non-epileptic events such as convulsive syncope were excluded [13]. Those recruited were followed for a minimum of 12 months. Data regarding seizure recurrence, their potential provoking factors, and use of ASM were collected by clinic review, telephone interview, and examination of medical records. Initiation of ASM was at the clinicians’ discretion. Follow-up start date was the time of the index seizure - the seizure that prompted a referral to the hospital [11].

*FIRST seizure trial group (FIRST)*: The FIRST [14], [15], [16] dataset comprised participants from a multi-centre randomised clinical trial comparing immediate and deferred ASM after a first unprovoked tonic-clonic seizure. Between February 1988 and February 1991, people aged two years or more seen within seven days after a witnessed first unprovoked tonic clonic seizure at one of 35 Italian academic clinics and hospitals were considered for recruitment. Those eligible were randomised within centre, by telephone call, to immediate treatment or to treatment in case of seizure recurrence. According to the clinician's preference, participants assigned to treatment were started on monotherapy with either carbamazepine, phenytoin, phenobarbital, or sodium valproate. All were followed up for at least six years. Follow-up start date was date of randomisation.

*Participant consents:* This was a re-analysis of randomised controlled trial and observational study data not requiring ethical approval.

### 2.2 Model development & internal validation

The outcome, time to seizure recurrence, was estimated for each individual with observations censored at date of last follow-up if no recurrence had occurred or if an individual was lost to follow-up, including death. Acute symptomatic and provoked seizures were not considered as a seizure recurrence. Analysis used Cox proportional hazards modelling on a time since entry timescale. Further details of the statistical methodology can be found in the Supplementary Material.

The list of eleven prognostic factors for potential inclusion in the model was: age at first seizure, gender, cause of seizure (remote symptomatic or not), neurological deficit, previous acute symptomatic seizure(s), epilepsy in first degree relative, seizure type, seizures only while asleep, electroencephalogram (EEG) result, computerised tomography (CT) or magnetic resonance imaging (MRI) scan result, and treatment policy. These were chosen based on clinical consensus and knowledge from previous predictive studies in epilepsy [7,17].

Individuals were classified as remote symptomatic if on entry to MESS the clinician considered that their seizure was caused by a past condition or event such as a head injury; meningitis or encephalitis; intracranial surgery; or other. Investigations (EEG and CT/MRI) were only undertaken if considered clinically indicated. Neurological deficit included hemiparesis and learning difficulty. An abnormal EEG was defined as focal or generalised slowing, or epileptiform abnormalities. CT/MRI scans were classified as abnormal or not. The continuous variable for age was investigated using fractional polynomial transformations [18], [19], [20], [21]. Missing data was rare and thus complete case analysis was undertaken whereby individuals with any missing data for any covariate (41 (3%) of all individuals) were removed from the analysis.

The performance of the model was evaluated in terms of discrimination and calibration. Discrimination was measured using Harrell's c-statistic [22] while calibration was reported graphically using calibration plots at one and three years after randomisation, within deciles of risk [23]. The developed model was validated internally and the model optimism was estimated. The predictor effects in the final developed model were penalised in order to account for this optimism [24].

### 2.3 External validation

For assessment of model transportability, the final optimism-adjusted model was externally validated in the NGPSE, WA and FIRST datasets. Validation was approached on a case-by-case basis to account for differences in predictor specification across the external datasets. In particular, when a predictor in the final model was not available in the external dataset (see Table 1), a reduced version of the final model was developed.

**Table 1:** Demographic summary of people in MESS, NGPSE, WA and FIRST – numbers are *n* (%), unless otherwise stated. Variables in bold signify those included in the developed multivariable model.

Variable	MESS(*n* = 1443)	NGPSE(*n* = 375)	WA(*n* = 1314)	FIRST(*n* = 419)
**Neurological deficit:** **Absent** **Present** **Missing**	**1311 (91)** **92 (6)** **40 (3)**	**Not reported**	**1018 (77)** **296 (23)** **-**	**376 (90)** **43 (10)** **-**
**Seizures:** **Focal** **Generalised** **Other** **Missing**	**557 (39)** **838 (58)** **30 (2)** **18 (1)**	**146 (39)** **109 (29)** **120 (32)** **-**	**636 (48)** **678 (52)** **-** **-**	**71 (17)** **348 (83)** **-** **-**
**EEG result:** **Normal** **Abnormal** **Not clinically indicated**	**540 (37)** **791 (55)** **112 (8)**	**78 (21)** **107 (28)** **190 (51)**	**654 (50)** **614 (47)** **46 (4)**	**180 (43)** **239 (57)** **-**
**CT or MRI scan result:** **Normal** **Abnormal** **Not clinically indicated**	**913 (63)** **123 (9)** **407 (28)**	**62 (17)** **50 (13)** **263 (70)**	**698 (53)** **596 (45)** **20 (2)**	**348 (83)** **71 (17)** **-**
**Treatment Policy:** **Immediate** **Delayed** **Missing**	**722 (50)** **721 (50)** **-**	**102 (27)** **273 (73)** **-**	**343 (26)** **970 (74)** **1 (0)**	**215 (51)** **204 (49)** **-**
Age at first seizure (years), median (IQR) [range]	24.3 (16.5, 42.3) [0.4–92.8]	36.8 (14.4, 62.7) [0.3, 62.7]	38.0 (24.0, 54.0) [14.0, 91.0]	22.0 (15.0, 36.0) [2.0, 76.0]
Gender: Male Female	826 (57) 617 (43)	186 (50) 189 (50)	827 (63) 487 (37)	236 (56) 183 (44)
Previous acute seizures: Febrile Other None Missing	105 (7) 27 (2) 1311 (91) -	Not reported	40 (3) - 1253 (95) 21 (2)	87 (21) - 332 (79) -
Epilepsy in 1st degree relative: Yes No Missing	162 (11) 1264 (88) 17 (1)	38 (10) 337 (90) -	135 (10) 1137 (87) 42 (3)	57 (14) 362 (86) -
Seizures only while asleep: Yes No Missing	265 (18) 1159 (80) 19 (2)	62 (17) 313 (83) -	296 (23) 1015 (77) 3 (0)	Not reported
Cause of seizure: Remote symptomatic Not remote symptomatic	189 (13) 1254 (87)	183 (49) 192 (51)	413 (31) 901 (69)	27 (6) 392 (94)
Seizure recurrence at: 1 year 3 years	489 (34) 624 (43)	129 (34) 156 (42)	578 (44) 666 (51)	109 (26) 152 (36)
Follow-up from entry (years), median (IQR)	4.5 (4.3, 4.7)	7.1 (7.0, 7.4)	5.7 (5.1, 6.4)	3.0 (2.8, 3.1)

#### 2.3.1 Model recalibration

Where calibration plots showed systematic under- or over-prediction of risk of recurrence in the external validation, recalibration via baseline hazard updating was undertaken to account for the different risk profile of the external dataset [25].

## 3 Results

**Fig. 1:**
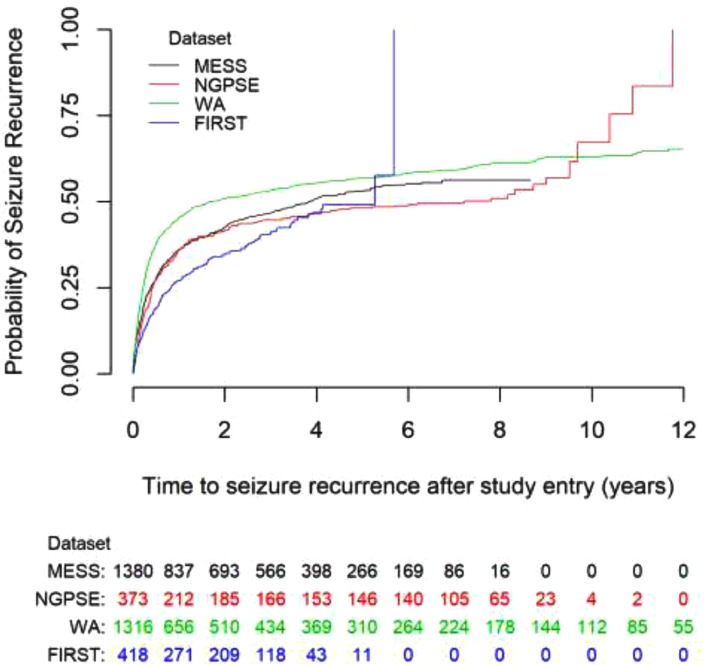
. Kaplan-Meier curve for time to seizure recurrence after randomisation with numbers at risk.

Table 1 provides a demographic summary of people in MESS, NGPSE, WA and FIRST. The Kaplan-Meier plot for seizure recurrence according to each dataset can be seen in Fig. 1. Within Table 1, focal seizure types are simple partial, complex partial, and simple or complex partial with secondary generalised tonic-clonic seizures. Generalised seizure types are myoclonic, typical absence, atypical absence, and tonic-clonic with neither aura nor definite focal onset.

### 3.1 Model development & internal validation

The parsimonious multivariable model (after backwards selection) for risk of seizure recurrence after study entry included covariates for neurological deficit, seizure type, EEG result, CT/MRI scan result and treatment policy. The optimism-adjusted c-statistic for the model was 0.575 and the calibration slope, adjusted for model fitting optimism, was 0.827. Optimism adjusted results for the predictors can be seen in Table 2. Specifically, people with neurological deficit were more likely to have seizure recurrence than those without. People with focal seizures were more likely to have a recurrence than those with generalised epilepsy, and those with an abnormal EEG were more likely to have a recurrence than those with a normal EEG result. People who were not clinically indicated for a CT or MRI scan were more likely to have a recurrence than those with a normal scan result, and those treated immediately after randomisation were less likely to have a seizure recurrence, after adjusting for the other covariates.

**Table 2:** Parsimonious multivariable model for risk of seizure recurrence after study entry according to the MESS dataset (adjusted for shrinkage).

**Variable**		**Hazard Ratio (95% CI)**
Neurological deficit	Absent Present	1.00 1.39 (1.09, 1.77)
Seizure Type	Generalised Focal Other	1.00 1.15 (1.01, 1.31) 0.80 (0.46, 1.38)
EEG result	Normal Abnormal Not clinically indicated	1.00 1.30 (1.13, 1.49) 1.18 (0.88, 1.57)
CT or MRI scan result	Normal Abnormal Not clinically indicated	1.00 1.11 (0.88, 1.40) 1.24 (1.08, 1.43)
Treatment Policy	Delayed Immediate	1.00 0.74 (0.65, 0.84)

The baseline estimates of seizure recurrence at one and three years were 0.351 and 0.462 once adjusted for shrinkage. Unadjusted estimates for all analyses can be found in the Supplementary Material. There was no evidence to doubt the proportional hazards assumption, except for treatment (*p*-values for Schoenfeld residual test – neurological deficit: 0.470, focal seizures: 0.049, other seizure types: 0.212, abnormal EEG: 0.714, EEG not clinically indicated: 0.971, abnormal CT: 0.838, CT not clinically indicated: 0.796, treatment policy: 0.019, overall: 0.141). The *p*-value for treatment of 0.02 provides some evidence that the effect of baseline treatment allocation changes over time. With the evidence being borderline, however, it is preferable to fit a common time effect for treatment for simplicity.

### 3.2 External validation

There were some differences in clinical characteristics across the datasets (Table 1). Participants in NGPSE and WA tended to be older than those in MESS, the latter reflecting the inclusion only of adults, and there were more females in NGPSE, although the variables for age and gender were not included in the parsimonious multivariable model. Considerably more people in NGPSE and WA were classified as having a remote symptomatic aetiology than those in MESS or FIRST. This may be because clinicians may not randomise these individuals to a treatment trial on the basis of equipoise as they believe treatment is required. This may also be due to different inclusion criteria - FIRST included only partial seizures with secondary generalisation, thus excluding a large number of first partial seizures without generalisation, many of whom may have had a remote symptomatic aetiology. Again, this variable was not included in the parsimonious multivariable model.

More participants in the NGPSE cohort did not have an EEG or CT/MRI scan than in MESS, in part reflecting the era in which the study was undertaken. Additionally, the number of EEG abnormalities were lower in NGPSE than in the other datasets. The proportions delaying treatment is fairly consistent between the studies. The impact of immediate treatment is, however, likely to be rather different in the non-randomised studies where immediate treatment was likely to be informed by other factors considered to be indicators for high risk of future seizures. The Kaplan-Meier plots for all four datasets, Fig. 1, show WA has a higher risk of seizure recurrence than MESS whilst FIRST has a lower risk. The risk in NGPSE is similar to that in MESS, especially over the first two years.

Despite the differences in characteristics and follow-up it is plausible that the NGPSE, WA and FIRST datasets came from the same ‘super-population’ as MESS [26]. Any observed differences in clinical characteristics are likely to represent selection bias including clinical and reporting practice where, in many cases, the only similarity between individuals is that they have had a seizure.

#### 3.2.1 NGPSE

The parsimonious multivariable model for the matched MESS data (MESS1) included covariates for cause of seizure, seizure type, EEG result, CT/MRI scan result and treatment policy. The optimism-adjusted c-statistic and slope were 0.351 and 0.462, respectively. Results, adjusted for optimism, can be seen in Table 3. People with remote symptomatic aetiology were more likely to have a seizure recurrence than those without, after accounting for seizure type, EEG result, CT/MRI result and treatment policy. All other results were consistent with the original MESS model.

**Table 3:** Parsimonious multivariable model for risk of seizure recurrence after study entry according to the MESS dataset without neurological deficit in the pool of potential prognostic factors (MESS1; post-shrinkage).

**Variable**		**Hazard Ratio (95% CI)**		
		**MESS1**	**MESS2**	**MESS3**
Neurological deficit	Absent Present	Not reported	1.00 1.38 (1.08, 1.76)	1.00 1.41 (1.12, 1.79)
Cause of seizure	Not remote symptomatic Remote symptomatic	1.00 1.23 (1.02, 1.48)	Not included in the model	Not included in the model
Seizure Type	Generalised Focal Other	1.00 1.16 (1.02, 1.32) 0.79 (0.45, 1.36)	1.00 1.15 (1.01, 1.30) Not reported	1.00 1.13 (0.99, 1.29) Not reported
EEG result	Normal Abnormal Not clinically indicated	1.00 1.32 (1.15, 1.51) 1.17 (0.88, 1.57)	1.00 1.32 (1.15, 1.51) 1.15 (0.85, 1.55)	1.00 1.30 (1.14, 1.48) Not reported
CT or MRI scan result	Normal Abnormal Not clinically indicated	1.00 1.12 (0.89, 1.41) 1.24 (1.07, 1.43)	1.00 1.11 (0.88, 1.39) 1.24 (1.07, 1.43)	Not included in the model
Treatment Policy	Delayed Immediate	1.00 0.74 (0.65, 0.84)	1.00 0.75 (0.66, 0.85)	1.00 0.75 (0.66, 0.85)

**Fig. 2:**
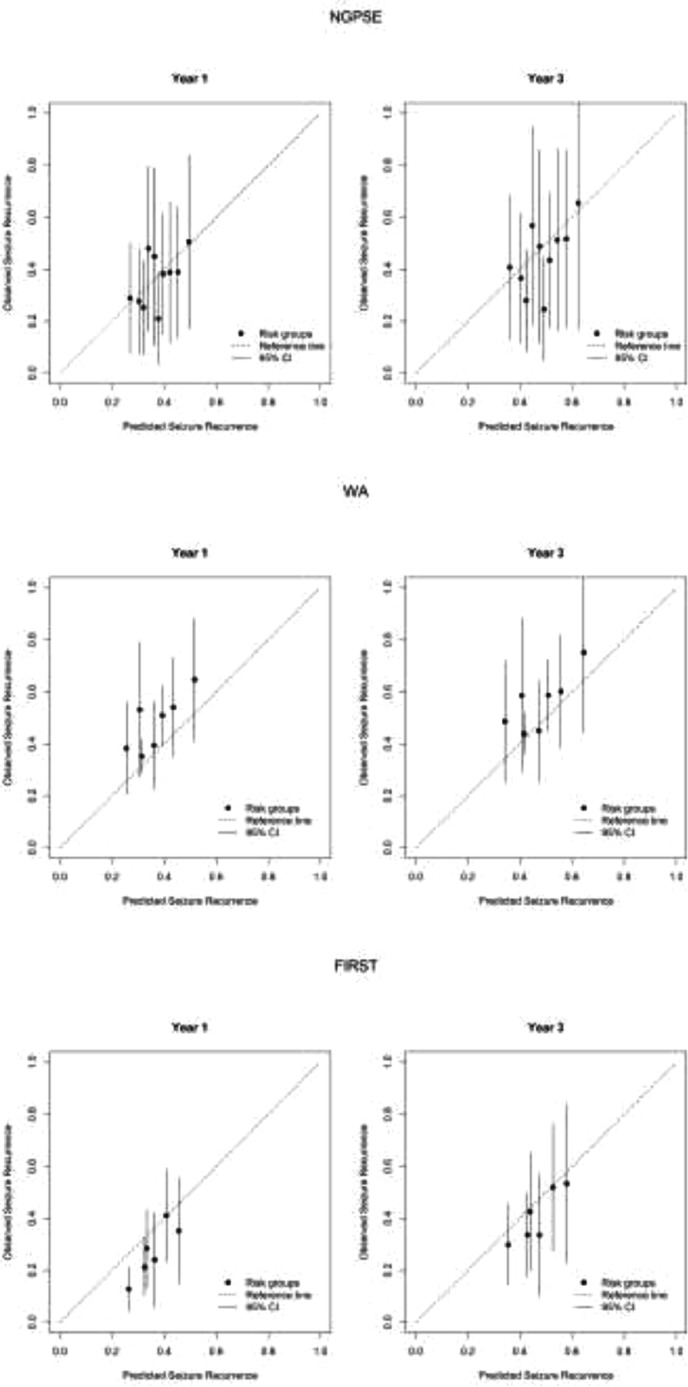
. Calibration plots comparing seizure recurrence in NGPSE to that predicted by MESS1, WA to that predicted by MESS2 and FIRST to that predicted by MESS3 at 1 and 3 years.

The c-statistic for the external validation of the MESS1 model in NGPSE data was 0.555. Calibration plots at one and three years can be seen in Fig. 2. As the points are all fairly closely clustered around the 45° line of agreement, the plots suggest that the MESS1 model is good at predicting risk of seizure recurrence at one and three years after randomisation.

#### 3.2.2 WA

The parsimonious multivariable model for the matched MESS data (MESS2) included covariates for neurological deficit, seizure type, EEG result, CT/MRI scan result and treatment policy. The optimism-adjusted c-statistic and slope were 0.576 and 0.825, respectively. Results, adjusted for optimism, can be seen in Table 3. All results were consistent with the original MESS model.

The c-statistic for the external validation of the MESS2 model in WA data was 0.558. Calibration plots at one and three years can be seen in Fig. 2. The plots suggest that MESS2 is under-predicting recurrence risk at one and three years as the points are all above the 45° line of agreement.

#### 3.2.3 FIRST

The parsimonious multivariable model for the matched MESS data (MESS3) included covariates for neurological deficit, seizure type, EEG result, and treatment policy. The optimism-adjusted c-statistic and slope were 0.574 and 0.834, respectively. Results, adjusted for optimism, can be seen in Table 3. All results were consistent with the original MESS model.

The c-statistic for the external validation was 0.597. Calibration plots at one and three years can be seen in Fig. 2. As the points are below the 45° line of agreement, the plots suggest that MESS3 is over-predicting risk of recurrence.

### 3.3 Model recalibration

**Fig. 3:**
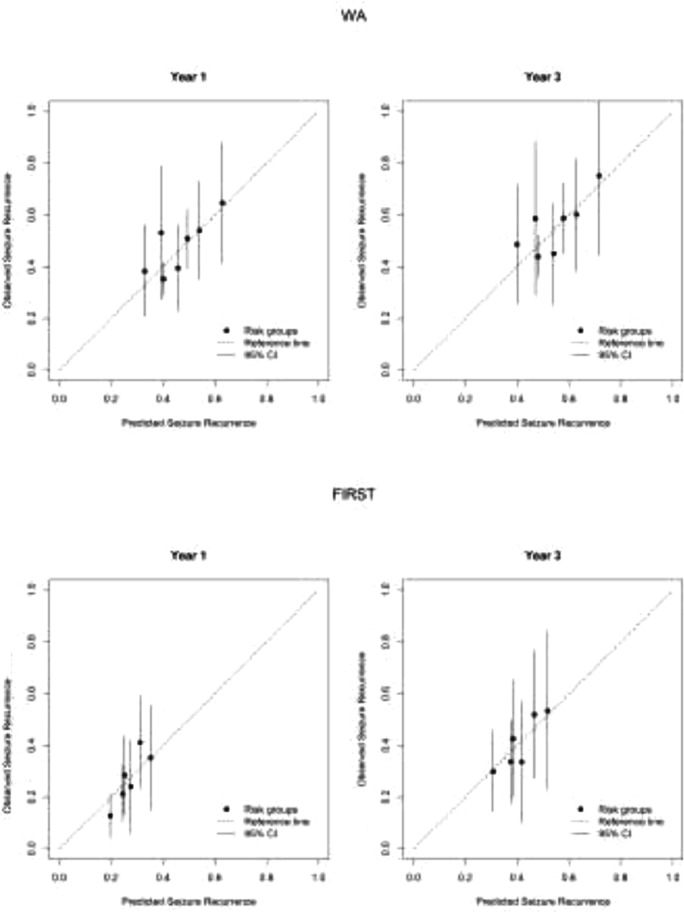
. Calibration plots comparing seizure recurrence in WA to that predicted by recalibrated MESS2 and in FIRST to that predicted by recalibrated MESS3.

The baseline estimates of seizure recurrence at one and three years in WA were 0.448 and 0.531, and 0.267 and 0.407 in FIRST, after adjusting for model-optimism. Calibration plots at one and three years for the recalibrated models can be seen in Fig. 3, respectively. As the points are now all fairly closely clustered around the 45° line of agreement, the plots suggest that the MESS2 and MESS3 models are good at predicting risk of seizure recurrence at one and three years after randomisation, once the underlying risk profile of participants in WA and FIRST is accounted for.

## 4 Discussion

People with neurological deficit, focal seizures, abnormal EEG results, not indicated for a CT/MRI scan result, or randomised to delayed treatment have a significantly higher risk of seizure recurrence. Not indicated for a CT/MRI scan maybe because of correlation with age, EEG or other factors. Indeed, in MESS, it is likely that age influenced whether someone had a scan. This may also reflect clinical practice specific to the UK.

The model was validated in three independent datasets from across the world. Variations to the original MESS model were required for all external datasets, so it is impossible to compare agreement between the datasets directly. Discrimination was fairly similar across all three external datasets, and close to 0.50, the value of no predictive value. It is, however, within keeping with other models in epilepsy and is indicative of the challenge of predicting seizures [27]. Calibration plots showed good agreement between observed and predicted probabilities in NGPSE. Plots for WA and FIRST showed less satisfactory agreement with the model generally under-predicting risk of seizure recurrence in WA, and over-predicting risk in FIRST until recalibration was undertaken to account for the differing underlying baseline survival probability in these datasets compared to MESS.

Many studies have evaluated risk of a second seizure after a first, and there have been several attempts to provide narrative summaries and meta-analyses of them [28,29]. Methodological weaknesses have been observed in many of the models and only one model considered people with single seizures or a new diagnosis of epilepsy [7]. That model identified number of seizures of all types at presentation, the presence of a neurological disorder, and an abnormal EEG as significant factors [7]. Number of seizures was not included in the model described here as the risk profile for people with a single seizure and people with early epilepsy are felt to be fairly consistent. Otherwise, the results are broadly in line with those of the previous model [7].

A prediction model for risk of seizure recurrence in people with a single seizure only, using MESS, has previously been developed and externally validated [5,6]. That model included covariates for aetiology, epilepsy in first degree relative, first seizure while sleep, EEG result, CT/MRI scan result and treatment policy. The model informed driving regulations in the UK and Europe, and is very similar to the model reported here. Differences are likely to be because of the population – in the driving model only people aged 16 years and over, with a first ever seizure, were considered whereas the current model includes people of all ages, including children, with newly diagnosed epilepsy and a first ever seizure. This will have increased the heterogeneity of the sample, but better reflects clinical practice.

The current study has limitations. The MESS dataset is over 10-years old but the prognosis of epilepsy has not changed over time. While the population of people with epilepsy has increased in age with more people who are elderly presenting, and these are under-represented in the MESS dataset, age was found not to be a predictor of outcome. MESS and FIRST were randomised trials and thus recruited people from unknown source populations. NGPSE is a population sample and WA is a tertiary hospital clinic-based sample. These different sources can have marked effects on the risk of recurrence, and on the models too as shown by the need to recalibrate the model for use in WA and FIRST. Additionally, no interaction terms were included in the model as it can be challenging to interpret interactions between continuous and categorical variables when a model is used within clinical practice. Also, information regarding adherence was not collected and therefore modelled. Medication adherence can interfere with outcome of seizures however we feel that our pragmatic approach of not modelling adherence reflects clinical practice where adherence can be challenging to measure.

Following the development and external validation of a model it is important to work with expert clinicians and patient and participant involvement groups to determine the most appropriate presentation method [30]. Once the presentation of the model has been finalised, it is then of interest to assess the model's clinical utility which evaluates whether the model leads to a positive change in clinical practice such as on prescribing practices and appointment management and/or in patients’ quality of life or behaviour [31]. Following these additional steps it should be possible to encourage the use of the prediction model within routine clinical practice. We plan to undertake these steps as an additional project.

## 5 Conclusion

Use of a clinical prediction model for risk of seizure recurrence after a single seizure or diagnosis of epilepsy can guide treatment decisions and counselling thus improving personalised management of people with seizures. The model developed here has been shown to perform well in independent data especially when recalibrated to account for differences in the underlying risk profile in two of the external datasets. The next steps include discussing the most appropriate presentation method for this model with end-users, testing the clinical utility of the model within clinical practice, and ultimately ensuring that this model is adopted as part of routine clinical practice to improve the lives of people with single seizures and early epilepsy.

## Role of the funding source

This article is independent research arising from a Post-Doctoral Fellowship (Dr Laura Bonnett - PDF-2015-08-044) supported by the National Institute for Health Research (https://www.nihr.ac.uk/). The views expressed in this publication are those of the authors and not necessarily those of the NHS, the National Institute for Health Research, or the Department of Health.

## Appendix Supplementary materials

Image, application 1

## References

[1] Hauser W.A., Beghi E. (2008). First seizure definitions and worldwide incidence and mortality. Epilepsia.

[2] Berg A.T., Shinnar S. (1991). The risk of seizure recurrence following a first unprovoked seizure: a quantitative review. Neurology.

[3] Marson A. (2005). Immediate versus deferred antiepileptic drug treatment for early epilepsy and single seizures: a randomised controlled trial. Lancet.

[4] Jacoby A. (2015). Quality-of-life outcomes of initiating treatment with standard and newer antiepileptic drugs in adults with new-onset epilepsy: findings from the SANAD trial. Epilepsia.

[5] Bonnett L.J. (2010). Risk of recurrence after a first seizure and implications for driving: further analysis of the multicentre study of early epilepsy and single seizures. Br Med J.

[6] Bonnett L.J. (2014). External validation of a prognostic model for seizure recurrence following a first unprovoked seizure and implications for driving. PLoS ONE.

[7] Kim L.G. (2006). Prediction of risk of seizure recurrence after a single seizure and early epilepsy: further results from the MESS trial. Lancet Neurol.

[8] Harrell F.E. (2001).

[9] Riley R.D. (2019).

[10] Hart Y.M. (1990). National general-practice study of epilepsy - recurrence after a first seizure. Lancet.

[11] Lawn N. (2015). Is the first seizure epilepsy—and when?. Epilepsia.

[12] Brown J.W. (2015). When is it safe to return to driving following first-ever seizure?. J Neurol Neurosurg Psychiatry.

[13] Berg A.T. (1999). Status epilepticus in children with newly diagnosed epilepsy. Ann Neurol Off J Am Neurol Assoc Child Neurol Soc.

[14] Musicco M. (1993). Randomized clinical-trial on the efficacy of antiepileptic drugs in reducing the risk of relapse after a 1st unprovoked tonic-clonic seizure. Neurology.

[15] Musicco M. (1997). Treatment of first tonic-clonic seizure does not improve the prognosis of epilepsy. Neurology.

[16] Leone M.A., Solari A., Beghi E. (2006). Treatment of the first tonic-clonic seizure does not affect long-term remission of epilepsy. Neurology.

[17] MacDonald B.K. (2000). Factors predicting prognosis of epilepsy after presentation with seizures. Ann Neurol.

[18] Royston P., Ambler G., Sauerbrei W. (1999). The use of fractional polynomials to model continuous risk variables in epidemiology. Int J Epidemiol.

[19] Royston P., Altman D.G. (1994). Regression using fractional polynomials of continuous covariates - parsimonious parametric modeling. Appl Stat J R Stat Soc Ser C.

[20] Royston P., Sauerbrei W. (2008).

[21] Royston P., Sauerbrei W. (2005). Building multivariable regression models with continuous covariates in clinical epidemiology–with an emphasis on fractional polynomials. Methods Inf Med.

[22] Harrell F.E., Lee K.L., Mark D.B. (1996). Multivariable prognostic models: issues in developing models, evaluating assumptions and adequacy, and measuring and reducing errors. Stat Med.

[23] Royston P., Altman D.G. (2013). External validation of a Cox prognostic model: principles and methods. BMC Med Res Methodol.

[24] Van Houwelingen J.C., Le Cessie S. (1990). Predictive value of statistical models. Stat Med.

[25] van Houwelingen H.C. (2000). Validation, calibration, revision and combination of prognostic survival models. Stat Med.

[26] Steyerberg E.W. (2009).

[27] Bonnett L.J. (2017). Breakthrough seizures—further analysis of the standard versus new antiepileptic drugs (SANAD) study. PLoS ONE.

[28] Berg A.T. (2008). Risk of recurrence after a first unprovoked seizure. Epilepsia.

[29] Rizvi S. (2017). Epidemiology of early stages of epilepsy: risk of seizure recurrence after a first seizure. Seizure.

[30] Bonnett L.J. (2019). Guide to presenting clinical prediction models for use in clinical settings. BMJ.

[31] Sachs M.C., Sjölander A., Gabriel E.E. (2020). Aim for clinical utility, not just predictive accuracy. Epidemiology.

