# Supplementary material for: Risk of seizure recurrence in people with single seizures and early epilepsy – Model development and external validation
Source: Seizure. 2022 Jan;94:26–32. doi: 10.1016/j.seizure.2021.11.007 (PMC8776562; doi:10.1016/j.seizure.2021.11.007)

Supporting Material

# Further Methodology

## Model Development

Backward selection according to Akaike’s Information Criterion was used to determine the parsimonious multivariable Cox proportional hazards model.[[17](#_ENREF_17)] Schöenfeld residuals [[18](#_ENREF_18)] and time-dependent covariate effects were used to investigate the proportional hazards assumption.

Discrimination indicates how well the model separates between individuals who have a recurrence from those that do not. Specifically, discrimination is the proportion of all pairs of individuals (excluding those where both are censored, who have the same event time, or one individual is censored before the other’s event time) in which the individual with the higher predicted seizure-free probability remained seizure-free longer than the other.[[22](#_ENREF_22)] Discrimination is assessed via the c-statistic. A c-statistic of 0.5 indicates no discrimination beyond chance, whereas a c-statistic of one indicates perfect discrimination. Calibration assesses if the predicted risks agree with the observed risks and is assessed via plots.

## Internal Validation

Non-parametric bootstrapping was used to estimate optimism, and examine model stability. In each of 500 bootstrap samples, the entire modelling process, including predictor selection, was repeated and the apparent model performance (calibration and discrimination in the bootstrap sample) was compared with the performance in the original sample. The average optimism across all bootstrap samples was then used to calculate the optimism-adjusted c-statistic and optimism-adjusted calibration slope.[[24](#_ENREF_24)] The latter was used as the uniform shrinkage factor.

## External Validation

Validation was approached on a case-by-case basis to account for differences in predictor specification across the external datasets. In particular, when a predictor in the final model was not available in the external dataset (see Table 1), a reduced version of the final model was developed. Specifically, the available predictor effects were re-estimated using the development data excluding the missing variable(s), and adjusted for optimism according to the described methods. These reduced models were then validated in the relevant external dataset. Again, discrimination was assessed via Harrell’s c-statistic and calibration was examined graphically, at one and three years after randomisation.

Specifically, neurological deficit was not reported in NGPSE so the pool of potential prognostic factors for MESS1 excluded neurological deficit. Other seizure types were not reported in WA. Therefore, people with other seizure types were removed from the dataset to develop MESS2. Seizure type, EEG and CT/MRI were categorised differently in FIRST than in MESS; other seizure type, and EEG and CT/MRI ‘not clinically indicated’ were not reported in FIRST. Therefore, people with other seizure types were removed from the MESS dataset. Additionally, people not clinically indicated for EEG or for CT/MRI in MESS were reclassified as having a normal EEG or CT/MRI. This modified dataset was used to develop a model that could be validated in FIRST (MESS3).

# Results not adjusted for optimism

## Model Development

Table e-1: Parsimonious multivariable model for risk of second seizure
after a first according to the variations on the MESS dataset (pre-shrinkage)

| **Variable** | | **Hazard Ratio**  **(95% CI)** | | | |
| --- | --- | --- | --- | --- | --- |
|  |  | **MESS** | **MESS1**  **(to match NGPSE)** | **MESS2**  **(to match WA)** | **MESS3**  **(to match FIRST)** |
| Neurological deficit | Absent  Present | 1.00  1.49 (1.10, 2.00) | Not included | 1.00  1.48 (1.10, 1.99) | 1.00  1.52 (1.15, 2.01) |
| Cause of seizure | Not remote symptomatic  Remote symptomatic | Not included | 1.00  1.28 (1.02, 1.60) | Not included | Not included |
| Seizure Type | Generalised  Partial  Other | 1.00  1.18 (1.01, 1.38)  0.76 (0.39, 1.48) | 1.00  1.19 (1.02, 1.39)  0.75 (0.39, 1.45) | 1.00  1.18 (1.01, 1.38)  Not reported | 1.00  1.16 (0.99, 1.35)  Not reported |
| EEG result | Normal  Abnormal  Not clinically indicated | 1.00  1.37 (1.16, 1.61)  1.22 (0.86, 1.73) | 1.00  1.39 (1.18, 1.64)  1.21 (0.85, 1.72) | 1.00  1.39 (1.18, 1.64)  1.18 (0.83, 1.69) | 1.00  1.37 (1.17, 1.60)  Not reported |
| CT or MRI scan result | Normal  Abnormal  Not clinically indicated | 1.00  1.13 (0.86, 1.50)  1.30 (1.10, 1.55) | 1.00  1.15 (0.87, 1.52)  1.30 (1.09, 1.54) | 1.00  1.13 (0.86, 1.49)  1.29 (1.09, 1.54) | Not included |
| Treatment Policy | Delayed  Immediate | 1.00  0.70 (0.60, 0.81) | 1.00  0.70 (0.60, 0.81) | 1.00  0.70 (0.60, 0.82) | 1.00  0.71 (0.61, 0.82) |
| *c-statistic* | | *0.589* | *0.584* | *0.591* | *0.587* |
| *Baseline estimate of seizure recurrence at 1 year* | | *0.349* | *0.349* | *0.350* | *0.351* |
| *Baseline estimate of seizure recurrence at 3 years* | | *0.459* | *0.459* | *0.460* | *0.461* |

## External Validation

Table e-2: c-statistics for the external validation

| **Dataset** | **c-statistic** |
| --- | --- |
| NGPSE | 0.555 |
| WA | 0.558 |
| FIRST | 0.597 |

Figure e-1: Calibration plots comparing seizure recurrence in NGPSE to that predicted by MESS1, in WA to that predicted by MESS2 and in FIRST to that predicted by MESS3 (pre-shrinkage)

**
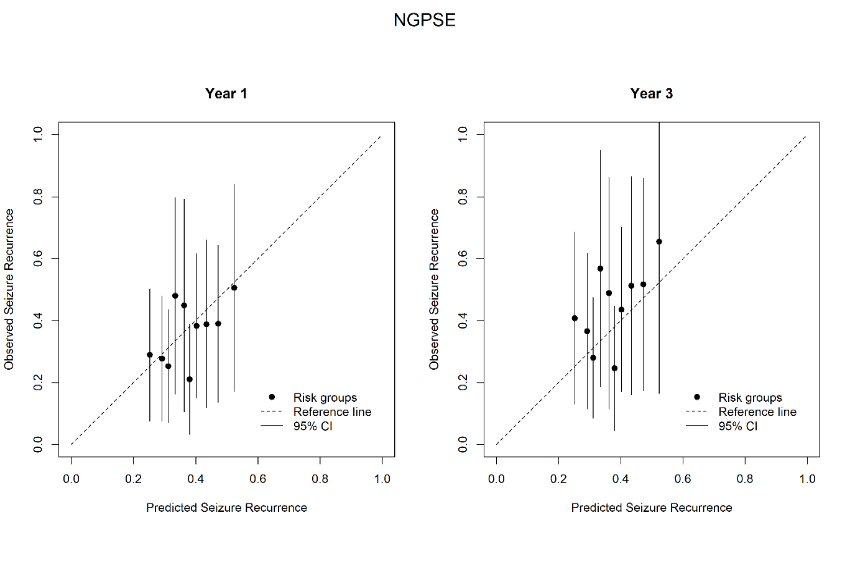
**


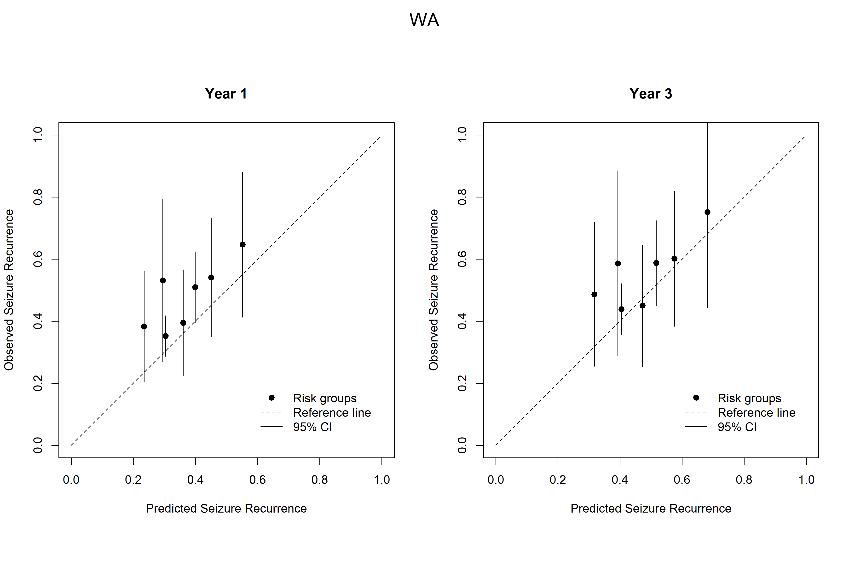


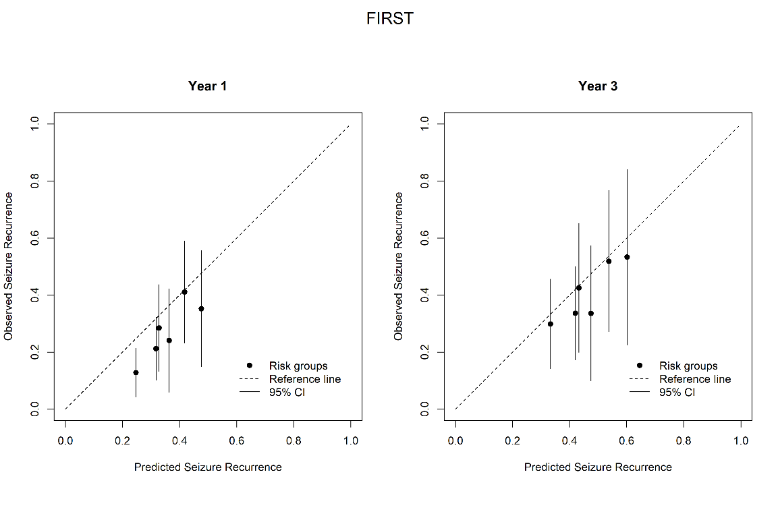


## Model Recalibration

Table e-3: c-statistics for the external validation

| **Dataset** | **Baseline estimates of seizure recurrence** | |
| --- | --- | --- |
|  | **1 year** | **3 years** |
| WA | 0.445 | 0.528 |
| FIRST | 0.265 | 0.405 |

Figure e-2: Calibration plots comparing seizure recurrence in WA to that predicted by recalibrated MESS2 and in FIRST to that predicted by recalibrated MESS3 (pre-shrinkage)


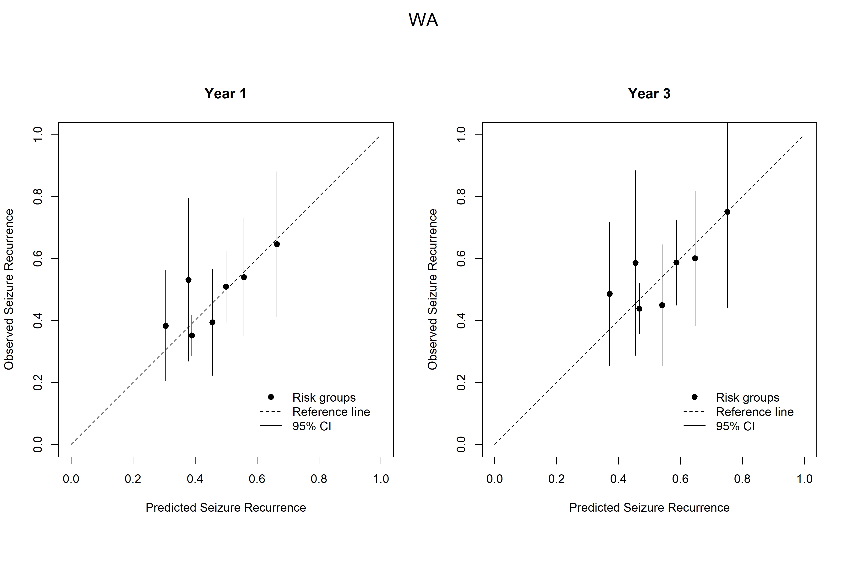


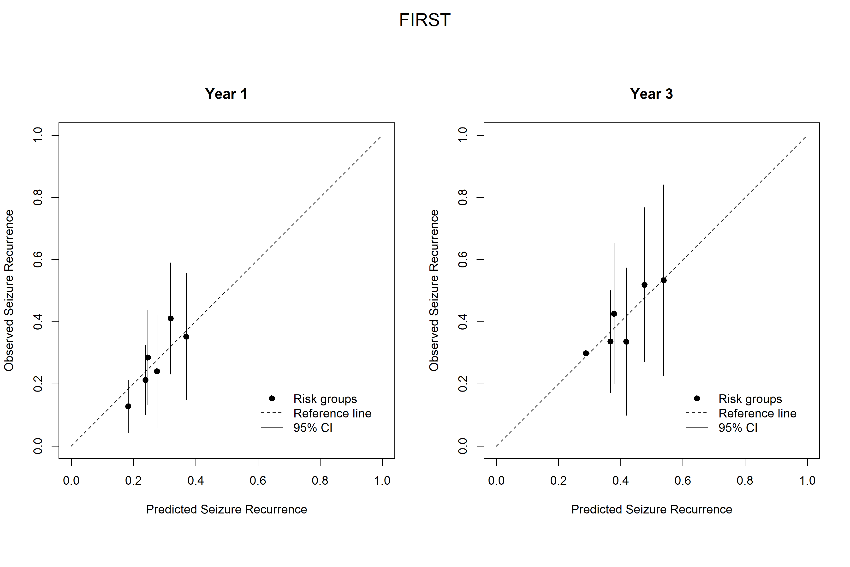

Supplement: Supplementary file 1 [file mmc1.docx]
